# The crystal structure of 5-(tri­fluoro­meth­yl)picolinic acid monohydrate reveals a water-bridged hydrogen-bonding network

**DOI:** 10.1107/S2056989020013523

**Published:** 2020-10-16

**Authors:** Naike Ye, Joseph M. Tanski

**Affiliations:** a Department of Chemistry, Vassar College, Poughkeepsie, NY 12604, USA

**Keywords:** crystal structure, hydrogen bonding, picolinic acid derivatives, tri­fluoro­methyl group

## Abstract

The title compound [systematic name: 5-(tri­fluoro­meth­yl)pyridine-2-carb­oxy­lic acid monohydrate], C_7_H_4_F_3_NO_2_·H_2_O, is the acid hydrate of a pyridine with a carb­oxy­lic acid group and a tri­fluoro­methyl substituent situated *para* to one another on the aromatic ring. The mol­ecule forms a centrosymmetric water-bridged hydrogen-bonding dimer with graph-set notation *R* ^4^_4_ (12). Hydrogen-bonding distances of 2.5219 (11) and 2.8213 (11) Å are observed between the donor carb­oxy­lic acid and the bridging water acceptor, and the donor water and carbonyl oxygen acceptor, respectively. The dimers are further linked into a two-dimensional sheet *via* two longer inter­molecular hydrogen-bonding inter­actions between the second hydrogen atom on the bridging water mol­ecule and both a pyridine nitro­gen atom and carbonyl oxygen with distances of 3.1769 (11) and 2.8455 (11) Å, respectively. The tri­fluoro­methyl groups extend out the faces of the sheet providing for F⋯F and C—H⋯F contacts between the sheets, completing the three-dimensional packing.

## Abstract 2

The title compound, [systematic name: 5-(tri­fluoro­meth­yl)pyridine-2-carb­oxy­lic acid monohydrate], C_7_H_4_F_3_NO_2_·H_2_O, is the acid hydrate of a pyridine with a carb­oxy­lic acid group and a tri­fluoro­methyl substituent situated *para* to one another on the aromatic ring. The mol­ecule forms a centrosymmetric water-bridged hydrogen-bonding dimer with graph-set notation 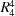 (12). The dimers are further linked into a two-dimensional sheet *via* two longer inter­molecular hydrogen-bonding inter­actions between the second hydrogen atom on the bridging water mol­ecule and both a pyridine nitro­gen atom and carbonyl oxygen. The tri­fluoro­methyl groups extend out the faces of the sheet providing for F⋯F and C—H⋯F contacts between the sheets, completing the three-dimensional packing.

## Chemical context   

Picolinic acids, pyridine derivatives with a carb­oxy­lic acid substituent at the 2-position, are common bidentate chelating agents of metallic elements in the human body (Grant *et al.*, 2009 ▸). The title compound, the hydrate of 5-(tri­fluoro­meth­yl)-2-pyridine­carb­oxy­lic acid (I), commonly known as 5-(tri­fluoro­meth­yl)picolinic acid, is a derivative of picolinic acid with potent chelating abilities and biological activities (Li *et al.*, 2019 ▸). Its transition-metal complexes also exhibit outstanding photophysical and electrochemical properties that make them promising phospho­rescent materials for OLEDs (Wei *et al.*, 2016 ▸). The compound may be synthesized from a range of synthetic routes, one of which relies on the carboxyl­ation reaction of 2-bromo-5-(tri­fluoro­meth­yl)pyridine with butyl­lithium (Cottet *et al.*, 2003 ▸).
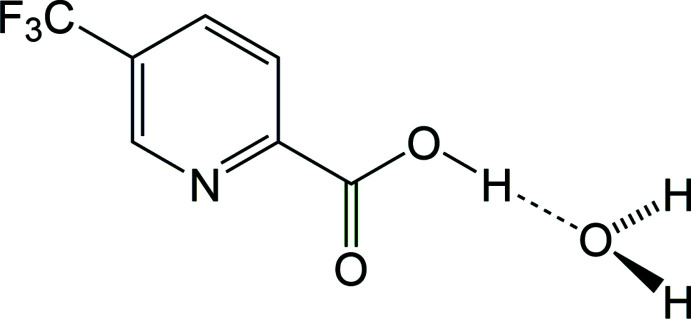


## Structural commentary   

**Figure 1:**
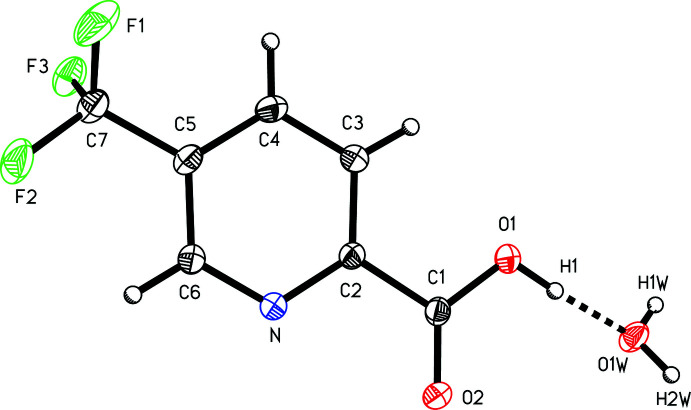
A view of 5-(tri­fluoro­meth­yl)picolinic acid (I) hydrate with the atom-numbering scheme. Displacement ellipsoids are shown at the 50% probability level.

The structure of 5-(tri­fluoro­meth­yl)picolinic acid (I) reveals that the crystalline material obtained from the supplier is a hydrate and confirms the position of the carb­oxy­lic acid group *ortho* to the pyridine nitro­gen atom with tri­fluoro­methyl substituent situated *para* to the acid group on the aromatic ring (Fig. 1 ▸). The two aromatic carbon–nitro­gen bonds have bond lengths of N—C2 of 1.3397 (12) Å and N—C6 of 1.3387 (12) Å, shorter than the aromatic C—C bonds, which have an average bond length of 1.387 (3) Å, a wedge-type motif typical in pyridine structures (Montgomery *et al.*, 2015 ▸). The aromatic carb­oxy­lic acid substituent has a C1—C2 bond length of 1.5081 (13) Å, similar to that of the tri­fluoro­methyl substituent C5—C7 of 1.5019 (13) Å, and the C—F bond lengths of the tri­fluoro­methyl group have an average bond length of 1.335 (4) Å. The carb­oxy­lic acid group is co-planar with the aromatic pyridine ring, with least-squares planes at an angle of 1.8 (2)°.

## Supra­molecular features   

The structure of 5-(tri­fluoro­meth­yl)picolinic acid (I) reported is a hydrate (Fig. 1 ▸) exhibiting a water-linked two-dimensional hydrogen-bonding network. Four different hydrogen-bonding inter­actions are observed between the picolinic acid and water mol­ecule, which acts as both a hydrogen-bonding donor and acceptor with the carb­oxy­lic acid group and pyridine nitro­gen atom (Table 1 ▸).

**Figure 2:**
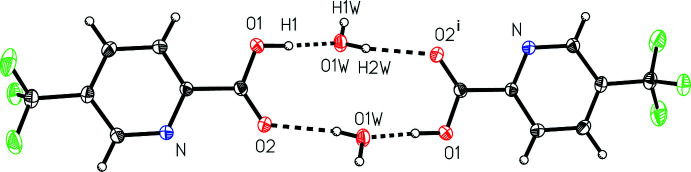
A view of the inter­molecular water-bridged hydrogen-bonding dimer in 5-(tri­fluoro­meth­yl)picolinic acid (I) hydrate.

The mol­ecular packing in the solid state can be characterized by the 5-(tri­fluoro­meth­yl)picolinic acid (I) hydrate asymmetric unit first forming a centrosymmetric water-bridged dimer unit with graph-set notation 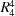 (12) (Fig. 2 ▸). The carb­oxy­lic acid hydrogen atom and the water oxygen form the stronger hydrogen bond, with an O1⋯O1*W* distance of 2.5219 (11) Å characterizing the O1—H1⋯O1*W* hydrogen bond, while the water hydrogen atom H2*W* bonds to the carbonyl oxygen atom with an O1*W*⋯O2^i^ distance of 2.8213 (11) Å charaterizing the O1*W*-*–*H2*W*⋯O2^i^ hydrogen bond [symmetry code: (i) −*x* + 1, −*y* + 1, −*z* + 2].

**Figure 3:**
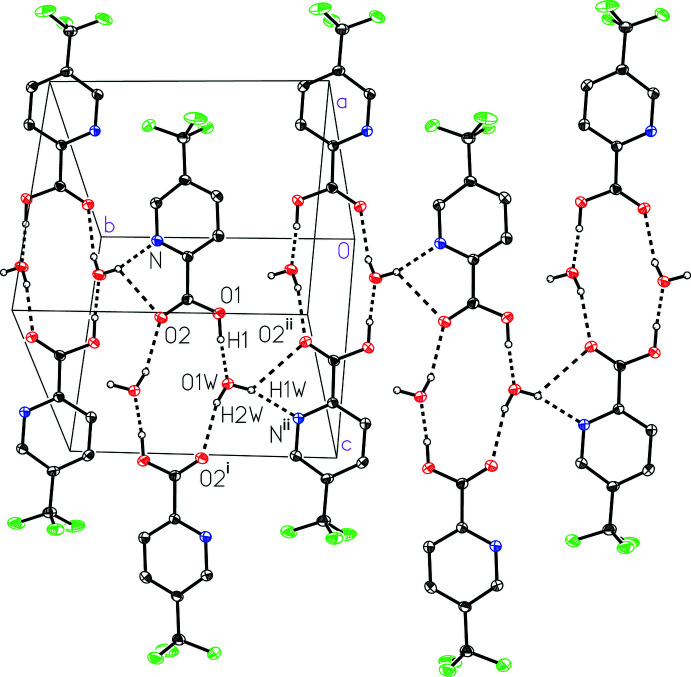
A view of a pleated strip formed between the water-bridged hydrogen-bonding dimers in 5-(tri­fluoro­meth­yl)picolinic acid (I) hydrate.

**Figure 4:**
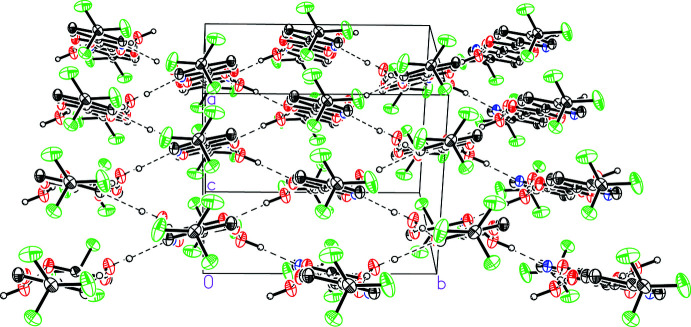
A view of the sheet hydrogen-bonding network in 5-(tri­fluoro­meth­yl)picolinic acid (I) hydrate viewing the water-bridge hydrogen-bonding dimers end-on shows the hydrogen-bonding inter­actions between the pleated strips forming a two-dimensional sheet.

**Figure 5:**
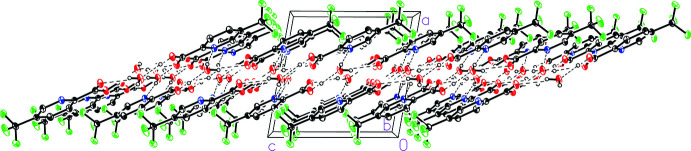
An edge-on view of the two-dimensional sheet formed between the water-bridged hydrogen-bonding dimers in 5-(tri­fluoro­meth­yl)picolinic acid (I) hydrate.

The water mol­ecules, specifically using the other water hydrogen atom H1*W*, further bridge the dimer units together to form a pleated strip or tape motif that propagates along the crystallographic [010] direction (Fig. 3 ▸). The the O1*W*⋯N^ii^ and O1*W*⋯O2^ii^ distances of 3.1769 (11) and 2.8455 (11) Å, respectively, characterize the O1*W*—H1*W*⋯N^ii^ and O1*W*—H1*W*⋯O2^ii^ hydrogen bonds [symmetry code: (ii) −*x* + 1, *y* − 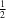, −*z* + 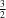]. The pleated nature of the strip exposes the H1*W* hydrogen atom of every other dimer to a pyridine nitro­gen in the strip adjacent to it, forming a thick two-dimensional sheet (Fig. 4 ▸). The sheet can be considered a bilayer with a hydro­philic core due to the presence of water mol­ecules and strong hydrogen bonding in the center and the more hydro­phobic tri­fluoro­methyl­aromatic groups extending to the faces of the sheet (Fig. 5 ▸).

**Figure 6:**
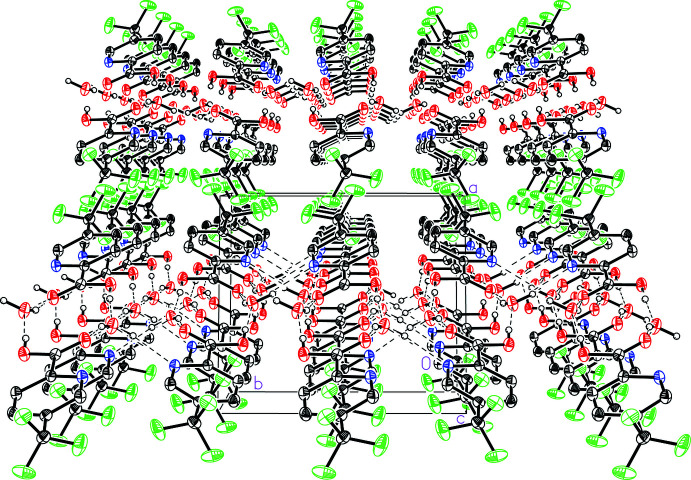
A view of the stacking of the two-dimensional sheets in 5-(tri­fluoro­meth­yl)picolinic acid (I) hydrate showing the tri­fluoro­methyl­aromatic inter­actions at the inter­faces of the sheets.

The sheets stack in the [100] direction (Fig. 6 ▸). The forces that guide the inter­molecular inter­actions between neighboring sheets are van der Waals forces including F⋯F and C—H⋯F contacts. The shortest weak C_ar­yl_—H⋯F inter­action, C4—H4*A*⋯F2 exhibits an H⋯F distance of 2.495 (1) Å. The most notable inter­action is a dimeric F⋯F inter­action between CF_3_ groups on neighboring sheets with an F1⋯F3 distance of 3.077 (1) Å, which is ∼0.15 Å longer than the sum of the van der Waals radii of fluorine (Bondi, 1964 ▸).

## Database survey   

Mono­carb­oxy­lic derivatives of pyridine, pyridine­carb­oxy­lic acids, are also commonly known as picolinic acid, nicotinic acid, or isonicotinic acid when the carboxyl group resides at the 2-, 3-, or 4- position, respectively. The Cambridge Structural Database (Version 5.40, update of March 2020; Groom *et al.*, 2016 ▸) contains no isomers of tri­fluoro­methyl-substituted pyridine­carb­oxy­lic acids. The crystal structure of the base of the title compound, picolinic acid (PICOLA02), was shown to be 1:1 co-crystals of its neutral and zwitterionic forms, where the nitro­gen atom can both be protonated and deprotonated (Hamazaki *et al.*, 1998 ▸). The inter­actions form a zigzag chain by N—H⋯N and O—H⋯O inter­molecular hydrogen bonding. A zwitterionic hydrogen-bonding motif can be found in substituted picolinic acid derivatives as well, such as 3-thioxo-2-pyridine­carb­oxy­lic acid (MPYDCX01; Bourne & Taylor, 1983 ▸).

A related solvated picolinic acid crystal structure can be found in the crystal structure of picolinic acid peroxosolvate (ANINES) which, while zwitterionic, exhibits a solvate-linked hydrogen-bonding pattern (Medvedev *et al.*, 2013 ▸). In this structure, every hydrogen peroxide mol­ecule links three picolinic acid mol­ecules together with two hydrogen bonds between the H_2_O_2_ hydrogen atoms and two carboxyl­ate groups, and an N—H⋯O hydrogen bond between the protonated pyridine nitro­gen atom and one oxygen atom of the H_2_O_2_ mol­ecule.

5-(Tri­fluoro­meth­yl)-2-pyridine­carb­oxy­lic acid has been used as a monoanionic ligand in several metal complexes, including with Co^II^ (VOVZOY; Li *et al.*, 2019 ▸), Cr^III^ (QEGWOR; Chai *et al.*, 2017 ▸), Mn^II^ (ROKSIW; Wang *et al.*, 2019 ▸), and Ir^III^ [COKGAN and COKGIV (Sanner *et al.*, 2019 ▸); GIZJOR (Hao *et al.*, 2019 ▸)]. While the Co^II^ and Mn^II^ complexes engage in inter­molecular hydrogen bonding with metal-coordinated water mol­ecules, the Cr^III^ complex contains a water of solvation that facilitates the formation of a hydrogen-bonding network. In a fashion reminiscent of 5-(tri­fluoro­meth­yl)picolinic acid (I) hydrate itself, [Cr(5-(tri­fluoro­meth­yl)picolinate)_2_(H_2_O)_2_]NO_3_·H_2_O hydrogen bonds into thick two-dimensional sheets with the tri­fluoro­methyl­aromatic groups extending to the faces of the sheets (Chai *et al.*, 2017 ▸).

## Synthesis and crystallization   

5-(Tri­fluoro­meth­yl)-2-pyridine­carb­oxy­lic acid (I, 95%) was purchased from Aldrich Chemical Company, USA, and was used as received.

## Refinement   

Crystal data, data collection and structure refinement details are summarized in Table 2 ▸. All non-hydrogen atoms were refined anisotropically. Hydrogen atoms on carbon atoms were included in calculated positions and refined using a riding model with C—H = 0.95 Å and *U*_iso_(H) = 1.2*U*_eq_(C) of the aryl C-atoms. The position of the carb­oxy­lic acid and water hydrogen atoms were found in the difference map and refined freely.

## Analytical data   

^1^H NMR (Bruker Avance III HD 400 MHz, DMSO *d*_6_): *δ* 3.70 (*br s*, OH), 8.21 (*d*, 1H, C_ar­yl_ *H*, *J_ortho_* = 8.0 Hz), 8.37 (*dd*, 1H, C_ar­yl_ *H*, *J_ortho_* = 8.2 Hz, *J_meta_* = 2.0 Hz), 9.07 (*s*, 1H, C_ar­yl_ *H*). ^13^C NMR (^13^C{^1^H}, 100.6 MHz, DMSO *d*_6_): *δ* 123.23 (*q*, *C*F_3_, *J*_C–F_ = 272.8 Hz), 124.73 (*s*, *C*_ar­yl_ *H*), 127.30 (*q*, *C*_ar­yl_CF_3_, *J*_C–F_ = 32.7 Hz), 135.12 (*q*, *C*_ar­yl_H, *J*_C–F_ = 3.5 Hz), 146.24 (*q*, *C*_ar­yl_H, *J*_C–F_ = 3.8 Hz), 151.98 (*s*, *C*_ar­yl_COOH), 165.13 (*s, C*OOH). ^19^F NMR (^19^F{^1^H}, 376.5 MHz, DMSO *d_6_*): *δ* −61.35 (*s*, 3F, C*F*3). IR (Thermo Nicolet iS50, FT–IR, KBr pellet, cm^−1^): 3469 (*s br*, O—H *str*), 3050 (*s*, C_ar­yl_—H *str*), 2849 (*w*), 2571 (*w*), 1961 (*m*), 1707 (*s*, C=O *str*), 1606 (*s*), 1582 (*s*), 1493 (*s*), 1440 (*s*), 1392 (*s*), 1328 (*m*), 1290 (*m*), 1251 (*s*), 1163 (*m*), 1126 (*s*), 1075 (*s*), 1023 (*s*), 948 (*s*), 878 (*m*), 864 (*s*), 806 (*s*), 760 (*s*), 704 (*s*), 643 (*s*), 524 (*s*). GC–MS (Agilent Technologies 7890A GC/5975C MS): *M^+^* = 191 amu, corresponding to the anhydrous form, 5-(tri­fluoro­meth­yl)pyridine-2-carb­oxy­lic acid (I), whose calculated exact mol­ecular ion mass is 191.02 amu.

## Supplementary Material

Crystal structure: contains datablock(s) global, I. DOI: 10.1107/S2056989020013523/dj2015sup1.cif

Structure factors: contains datablock(s) I. DOI: 10.1107/S2056989020013523/dj2015Isup2.hkl

Supporting information file. DOI: 10.1107/S2056989020013523/dj2015Isup3.cml

CCDC reference: 2036131

Additional supporting information:  crystallographic information; 3D view; checkCIF report

## Figures and Tables

**Table 1:** Hydrogen-bond geometry (Å, °)

*D*—H⋯*A*	*D*—H	H⋯*A*	*D*⋯*A*	*D*—H⋯*A*
O1—H1⋯O1*W*	0.92 (2)	1.60 (2)	2.5219 (11)	174 (2)
O1*W*—H2*W*⋯O2^i^	0.808 (19)	2.038 (19)	2.8213 (11)	163.2 (17)
O1*W*—H1*W*⋯O2^ii^	0.859 (19)	2.615 (18)	3.1769 (11)	124.1 (13)
O1*W*—H1*W*⋯N^ii^	0.859 (19)	2.008 (18)	2.8455 (11)	164.5 (16)

Symmetry codes: (i) 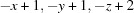; (ii) 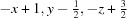.

**Table 2:** Experimental details

Crystal data	
Chemical formula	C_7_H_4_F_3_NO_2_·H_2_O
*M*_r_	209.13
Crystal system, space group	Monoclinic, *P*2_1_/*c*
Temperature (K)	125
*a*, *b*, *c* (Å)	8.9213 (10), 10.0759 (12), 9.1010 (11)
β (°)	99.983 (2)
*V* (Å^3^)	805.70 (16)
*Z*	4
Radiation type	Mo *K*α
μ (mm^−1^)	0.18
Crystal size (mm)	0.32 × 0.25 × 0.14

Data collection	
Diffractometer	Bruker APEXII CCD
Absorption correction	Multi-scan (*SADABS*; Bruker, 2017 ▸)
*T*_min_, *T*_max_	0.89, 0.98
No. of measured, independent and observed [*I* > 2σ(*I*)] reflections	19282, 2463, 2126
*R*_int_	0.026
(sin θ/λ)_max_ (Å^−1^)	0.715

Refinement	
*R*[*F* ^2^ > 2σ(*F* ^2^)], *wR*(*F* ^2^), *S*	0.034, 0.103, 1.07
No. of reflections	2463
No. of parameters	139
H-atom treatment	H atoms treated by a mixture of independent and constrained refinement
Δρ_max_, Δρ_min_ (e Å^−3^)	0.50, −0.26

Computer programs: *APEX2* and *SAINT* (Bruker, 2017 ▸), *SHELXT2014/5* (Sheldrick, 2015*a* ▸), *SHELXL2017/1* (Sheldrick, 2015*b* ▸), *SHELXTL2014* (Sheldrick, 2008 ▸), *OLEX2* (Dolomanov *et al.*, 2009 ▸), and *Mercury* (Macrae *et al.*, 2020 ▸).

## supplementary crystallographic information

### Crystal data

**Table:**

C_7_H_4_F_3_NO_2_·H_2_O	*F*(000) = 424
*M**_r_* = 209.13	*D*_x_ = 1.724 Mg m^−^^3^
Monoclinic, *P*2_1_/*c*	Mo *K*α radiation, λ = 0.71073 Å
*a* = 8.9213 (10) Å	Cell parameters from 7786 reflections
*b* = 10.0759 (12) Å	θ = 3.0–30.2°
*c* = 9.1010 (11) Å	µ = 0.18 mm^−^^1^
β = 99.983 (2)°	*T* = 125 K
*V* = 805.70 (16) Å^3^	Block, colourless
*Z* = 4	0.32 × 0.25 × 0.14 mm

### Data collection

**Table:**

Bruker APEXII CCD diffractometer	2463 independent reflections
Radiation source: fine-focus sealed tube	2126 reflections with *I* > 2σ(*I*)
Graphite monochromator	*R*_int_ = 0.026
Detector resolution: 8.3333 pixels mm^-1^	θ_max_ = 30.5°, θ_min_ = 2.3°
φ and ω scans	*h* = −12→12
Absorption correction: multi-scan (SADABS; Bruker, 2017)	*k* = −14→14
*T*_min_ = 0.89, *T*_max_ = 0.98	*l* = −13→13
19282 measured reflections	

### Refinement

**Table:**

Refinement on *F*^2^	Primary atom site location: dual
Least-squares matrix: full	Secondary atom site location: difference Fourier map
*R*[*F*^2^ > 2σ(*F*^2^)] = 0.034	Hydrogen site location: mixed
*wR*(*F*^2^) = 0.103	H atoms treated by a mixture of independent and constrained refinement
*S* = 1.07	*w* = 1/[σ^2^(*F*_o_^2^) + (0.0559*P*)^2^ + 0.2301*P*] where *P* = (*F*_o_^2^ + 2*F*_c_^2^)/3
2463 reflections	(Δ/σ)_max_ < 0.001
139 parameters	Δρ_max_ = 0.50 e Å^−^^3^
0 restraints	Δρ_min_ = −0.26 e Å^−^^3^

### Special details

**Table:**

Geometry. All esds (except the esd in the dihedral angle between two l.s. planes) are estimated using the full covariance matrix. The cell esds are taken into account individually in the estimation of esds in distances, angles and torsion angles; correlations between esds in cell parameters are only used when they are defined by crystal symmetry. An approximate (isotropic) treatment of cell esds is used for estimating esds involving l.s. planes.

### Fractional atomic coordinates and isotropic or equivalent isotropic displacement parameters (Å^2^)

**Table:**

	*x*	*y*	*z*	*U*_iso_*/*U*_eq_	
F1	1.02797 (8)	0.44945 (10)	0.19056 (8)	0.0387 (2)	
F2	0.91945 (11)	0.63911 (8)	0.13936 (9)	0.0398 (2)	
F3	0.80545 (8)	0.45876 (7)	0.06062 (7)	0.02763 (17)	
O1	0.62864 (9)	0.36976 (7)	0.76271 (8)	0.02126 (17)	
H1	0.572 (2)	0.365 (2)	0.838 (2)	0.056 (6)*	
O2	0.57174 (9)	0.58725 (7)	0.74769 (8)	0.02259 (17)	
N	0.69746 (10)	0.60686 (8)	0.49521 (9)	0.01815 (18)	
C1	0.62637 (11)	0.48809 (9)	0.70248 (10)	0.01643 (18)	
C2	0.70005 (11)	0.48977 (9)	0.56536 (10)	0.01547 (18)	
C3	0.76396 (12)	0.37599 (9)	0.51689 (11)	0.01936 (19)	
H3A	0.762813	0.29467	0.569519	0.023*	
C4	0.82968 (12)	0.38369 (10)	0.38964 (11)	0.0205 (2)	
H4A	0.87462	0.307748	0.353174	0.025*	
C5	0.82825 (11)	0.50451 (10)	0.31708 (11)	0.01741 (19)	
C6	0.76044 (12)	0.61375 (10)	0.37220 (11)	0.01917 (19)	
H6A	0.758767	0.695976	0.32085	0.023*	
C7	0.89493 (12)	0.51403 (10)	0.17683 (11)	0.0210 (2)	
O1W	0.46520 (10)	0.34230 (8)	0.96069 (9)	0.02433 (18)	
H2W	0.469 (2)	0.3734 (17)	1.043 (2)	0.040 (5)*	
H1W	0.4241 (19)	0.2651 (19)	0.9607 (19)	0.043 (5)*	

### Atomic displacement parameters (Å^2^)

**Table:**

	*U*^11^	*U*^22^	*U*^33^	*U*^12^	*U*^13^	*U*^23^
F1	0.0238 (4)	0.0669 (6)	0.0280 (4)	0.0091 (3)	0.0118 (3)	−0.0011 (4)
F2	0.0630 (5)	0.0278 (4)	0.0368 (4)	−0.0179 (3)	0.0317 (4)	−0.0058 (3)
F3	0.0315 (4)	0.0352 (4)	0.0167 (3)	−0.0061 (3)	0.0055 (2)	−0.0055 (3)
O1	0.0303 (4)	0.0167 (3)	0.0190 (3)	0.0011 (3)	0.0104 (3)	0.0034 (3)
O2	0.0328 (4)	0.0185 (3)	0.0189 (3)	0.0026 (3)	0.0112 (3)	−0.0006 (3)
N	0.0240 (4)	0.0154 (4)	0.0161 (4)	0.0013 (3)	0.0066 (3)	0.0005 (3)
C1	0.0192 (4)	0.0165 (4)	0.0135 (4)	−0.0014 (3)	0.0027 (3)	0.0004 (3)
C2	0.0180 (4)	0.0150 (4)	0.0136 (4)	−0.0004 (3)	0.0031 (3)	−0.0006 (3)
C3	0.0252 (5)	0.0151 (4)	0.0188 (4)	0.0012 (3)	0.0066 (4)	0.0006 (3)
C4	0.0248 (5)	0.0174 (4)	0.0207 (4)	0.0015 (3)	0.0078 (4)	−0.0033 (3)
C5	0.0182 (4)	0.0197 (4)	0.0153 (4)	−0.0028 (3)	0.0056 (3)	−0.0031 (3)
C6	0.0252 (5)	0.0167 (4)	0.0168 (4)	−0.0006 (3)	0.0070 (3)	0.0006 (3)
C7	0.0218 (5)	0.0234 (5)	0.0194 (4)	−0.0044 (4)	0.0079 (4)	−0.0036 (4)
O1W	0.0376 (4)	0.0188 (3)	0.0198 (4)	−0.0073 (3)	0.0140 (3)	−0.0031 (3)

### Geometric parameters (Å, º)

**Table:**

F1—C7	1.3402 (13)	C3—C4	1.3879 (13)
F2—C7	1.3335 (13)	C3—H3A	0.95
F3—C7	1.3317 (12)	C4—C5	1.3840 (14)
O1—C1	1.3109 (11)	C4—H4A	0.95
O1—H1	0.92 (2)	C5—C6	1.3908 (13)
O2—C1	1.2143 (12)	C5—C7	1.5019 (13)
N—C6	1.3387 (12)	C6—H6A	0.95
N—C2	1.3397 (12)	O1W—H2W	0.808 (19)
C1—C2	1.5081 (13)	O1W—H1W	0.859 (19)
C2—C3	1.3861 (13)		
			
C1—O1—H1	113.0 (13)	C4—C5—C6	119.52 (9)
C6—N—C2	117.98 (8)	C4—C5—C7	119.30 (9)
O2—C1—O1	125.78 (9)	C6—C5—C7	121.14 (9)
O2—C1—C2	121.95 (8)	N—C6—C5	122.17 (9)
O1—C1—C2	112.26 (8)	N—C6—H6A	118.9
N—C2—C3	123.42 (9)	C5—C6—H6A	118.9
N—C2—C1	115.48 (8)	F3—C7—F2	107.12 (9)
C3—C2—C1	121.09 (8)	F3—C7—F1	105.68 (8)
C2—C3—C4	118.41 (9)	F2—C7—F1	107.51 (9)
C2—C3—H3A	120.8	F3—C7—C5	112.15 (8)
C4—C3—H3A	120.8	F2—C7—C5	112.61 (8)
C5—C4—C3	118.49 (9)	F1—C7—C5	111.38 (9)
C5—C4—H4A	120.8	H2W—O1W—H1W	107.4 (16)
C3—C4—H4A	120.8		

### Hydrogen-bond geometry (Å, º)

**Table:**

*D*—H···*A*	*D*—H	H···*A*	*D*···*A*	*D*—H···*A*
O1—H1···O1*W*	0.92 (2)	1.60 (2)	2.5219 (11)	174 (2)
O1*W*—H2*W*···O2^i^	0.808 (19)	2.038 (19)	2.8213 (11)	163.2 (17)
O1*W*—H1*W*···O2^ii^	0.859 (19)	2.615 (18)	3.1769 (11)	124.1 (13)
O1*W*—H1*W*···N^ii^	0.859 (19)	2.008 (18)	2.8455 (11)	164.5 (16)

Symmetry codes: (i) −*x*+1, −*y*+1, −*z*+2; (ii) −*x*+1, *y*−1/2, −*z*+3/2.
